# Troponina I por Percentil 99 da Definição Universal de Infarto do Miocárdio versus Ponto de Corte de Melhor Acurácia em Síndromes Coronárias Agudas

**DOI:** 10.36660/abc.20210191

**Published:** 2022-04-26

**Authors:** Antonio Haddad Tapias, Gustavo Bernardes de Figueiredo Oliveira, João Italo Dias França, Rui Fernando Ramos

**Affiliations:** 1 Instituto Dante Pazzanese de Cardiologia São Paulo SP Brasil Instituto Dante Pazzanese de Cardiologia, São Paulo, SP – Brasil

**Keywords:** Troponin I, Acute Coronary Syndrome, Myocardial Revascularization

## Abstract

**Fundamento:**

O diagnóstico de síndrome coronária aguda (SCA) e a estratificação de risco contemporâneos são fundamentais para o manejo apropriado e redução da mortalidade e eventos isquêmicos recorrentes, tanto na fase aguda quanto após hospitalização. A Definição Universal de Infarto do Miocárdio recomenda a detecção de curva de troponina acima do limite superior do percentil 99.

**Objetivos:**

Avaliar a ocorrência de óbito e infarto agudo do miocárdio (IAM) na fase precoce em pacientes sem elevação de troponina (<0,034 ng/mL), pacientes com mínima elevação [acima do percentil 99 (>0,034 ng/mL e <0,12 ng/mL)], e pacientes com maiores elevações [acima do ponto de corte para IAM pelo kit utilizado (≥0,12 ng/mL)]; e avaliar o impacto dos níveis de troponina na indicação de estratégia invasiva e revascularização miocárdica.

**Métodos:**

Estudo de corte transversal de pacientes com SCA com avaliação do pico da troponina I, escores de risco, análise prospectiva de desfechos clínicos até 30 dias e testes bilaterais de significância, com nível de significância adotado sendo < 0,05.

**Resultados:**

Foram avaliados 494 pacientes com SCA. Troponina > percentil 99 e abaixo do ponto de corte, assim como valores maiores (acima do ponto de corte), foram associados à maior incidência do desfecho composto (p<0,01) e de revascularização percutânea ou cirúrgica (p<0,01), sem diferença significante em mortalidade até 30 dias.

**Conclusões:**

Valores de troponina elevados acima do percentil 99 pela Definição Universal de IAM apresentam papel prognóstico e agregam informação útil ao diagnóstico clínico e escores de risco na identificação de pacientes com maior probabilidade de benefício com estratificação invasiva e procedimentos de revascularização coronária.

## Introdução

As doenças cardiovasculares representam as principais causas de morte na população brasileira acima de 60 anos^1^ e, no contexto mundial, são importantes causas de invalidez, hospitalizações e óbito, principalmente em países com menor renda *per capita.*^2,3^

Em síndromes coronárias agudas (SCA), o eletrocardiograma (ECG) e a dosagem da troponina, realizados de forma seriada e associada ao exame clínico, são elementos essenciais para o diagnóstico e o manejo apropriados.^4^ Além de estabelecer o diagnóstico de infarto agudo do miocárdio (IAM) no contexto de isquemia miocárdica aguda, a dosagem da troponina é útil na estratificação de risco para estratégia invasiva.^5^ Além disso, o valor pico da troponina apresenta correlação com a extensão da necrose e com a fração de ejeção do ventrículo esquerdo (FEVE), importantes determinantes da mortalidade após IAM.^6,7^ Valores elevados de troponina também mostraram correlação com doença arterial coronariana (DAC) multiarterial e maior gravidade de estenose em pacientes com SCA sem supradesnivelamento do segmento ST (SCASSST),^8,9^ além de relação diretamente proporcional com taxa de desfechos clínicos.^10-13^ Entretanto, o nível pico de troponina parece ter menor importância relativa em pacientes submetidos a procedimentos de revascularização precoce.^14^

Fatores como a gravidade e a complexidade da DAC, o uso prévio de ácido acetilsalicílico e a precocidade da angiografia coronária estão associados ao pico da troponina na SCASSST.^15^ Na fase de estabilização clínica após SCA, troponina elevada está associada à maior mortalidade cardiovascular e por todas as causas, independente de covariáveis.^16^ Pacientes com troponina muito elevada apresentam DAC mais complexa e, por plausibilidade fisiopatológica, podem receber indicação de revascularização com maior frequência em comparação aos pacientes sem elevação da troponina. Por outro lado, alguns estudos não encontraram associação entre níveis altos de troponina e piores desfechos clínicos.^17,18^

### Níveis de troponina propostos para diagnóstico em SCA

Conforme recomendações da *International Federation of Clinical Chemistry* e da *National Academy of Clinical Biochemistry*, troponina elevada é definida acima do percentil 99 para uma população considerada saudável e com coeficiente de variação (CV) intraensaio < 10%,^19^ embora muitos kits de troponina apresentem imprecisão analítica com base nesse percentil.^20-22^ Portanto, para o uso da troponina no diagnóstico de IAM, é necessária uma curva ascendente e/ou descendente do biomarcardor, com pelo menos um valor acima do percentil 99, baseado na população de referência e conforme sexo, etnia, e outros fatores.^23,24^ Alguns estudos ressaltam a importância de uniformizar o nível de troponina para diagnóstico de IAM em laboratórios hospitalares para aprimorar a decisão clínica, individualizar o valor para população atendida naquele hospital, além de facilitar relatórios em estudos clínicos.^25,26^

Desse modo, os objetivos deste estudo foram avaliar a ocorrência de desfechos clinicamente relevantes (óbito, IAM, e desfecho composto) em pacientes com SCASSST na fase precoce; comparar três grupos divididos de acordo com respectivas faixas de mensurações de troponina I: sem elevação (<0,034 ng/mL, ou seja, abaixo do percentil 99), com mínima elevação [“Definição Universal de IAM”, acima do percentil 99 (>0,034 ng/mL e <0,12 ng/mL)], e com maior elevação [“Ponto de corte de maior acurácia” estipulado no kit local (≥0,12 ng/mL); e avaliar a associação entre os três grupos e a indicação de estratégia invasiva e de procedimentos de revascularização miocárdica na fase hospitalar. A hipótese é de que o nível do percentil 99, mesmo abaixo do valor de corte do kit de troponina I esteja associado a impacto clínico e maior indicação de estratificação invasiva e revascularização miocárdica, em comparação com níveis negativos, corroborando a proposta da definição universal para diagnóstico de IAM.

## Métodos

### Características do estudo e aspectos éticos

Estudo observacional, transversal, com seguimento até 30 dias para avaliação prospectiva de óbito, infarto, e desfecho composto em pacientes com SCASSST admitidos em Unidade Coronária (UCO), separados em grupos conforme nível de troponina. Todos os eventos clínicos foram pré-especificados e avaliados após coleta sistemática de informações em banco de dados, além das indicações de estratificação de risco invasivo ou não invasivo, tratamentos intra-hospitalares, e testes laboratoriais de rotina. Consentimento informado foi obtido por meio de termo de consentimento livre e esclarecido. Este estudo recebeu aprovação pelo Comitê de Ética em Pesquisa local em abril de 2019. O recrutamento dos pacientes ocorreu entre maio de 2019 e janeiro de 2020.

### Critérios de inclusão

• Idade ≥ 18 anos
•  Hospitalizados na UCO
•  Diagnóstico de SCASSST
• O diagnóstico de IAMSSST obedeceu a dois dos seguintes critérios:
- Quadro clínico sugestivo de SCA;
- ECG demonstrando depressão do segmento ST, inversão de ondas T ou achados inespecíficos.
- Curva de troponina, ascendente e/ou descendente, com pelo menos um valor acima de 0,12 ng/mL (valor para diagnóstico de IAM no kit de troponina utilizado no Instituto Dante Pazzanese de Cardiologia).

O reinfarto foi definido conforme recomendações da 4ª definição universal de IAM, sendo suspeito na presença de sinais ou sintomas de infarto, necessitando de nova dosagem de troponina. Um aumento de 20% nos níveis de troponina em pacientes já com elevação de troponina, ou um novo aumento de troponina em pacientes com exame previamente normal, confirmam o diagnóstico.

  • O diagnóstico de angina instável obedeceu a dois dos seguintes critérios:
- Quadro clínico sugestivo de SCA;
- ECG demonstrando depressão do segmento ST, inversão de ondas T ou achados inespecíficos.
- Ausência de troponina com valores acima de 0,034 ng/mL (referências para diagnóstico de IAM foram a Definição Universal de IAM^27^e Diretriz da Sociedade Europeia de Cardiologia^28^).

### Critérios de exclusão

- Não consentimento para participação no estudo
- Pacientes transferidos para realização de estratégia invasiva após 48h de manejo do episódio inicial de SCA em outro hospital.

### Variáveis analisadas

Foram avaliados dados demográficos, fatores de risco cardiovascular e comorbidades, medicações de uso prévio, parâmetros hemodinâmicos simples não invasivos, características da angiografia coronária (nos casos indicados para estratégia invasiva) e escores de risco GRACE e CRUSADE. Os testes laboratoriais, procedimentos e tratamentos na fase hospitalar seguiram protocolos da instituição.

### Teste de troponina cardíaca

**Figura 1:**
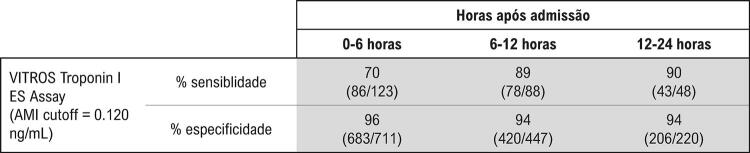
– Especificações do kit de troponina I.

O kit usado para dosagem de troponina I foi o kit de reagente imunodiagnóstico VITROS® da *Ortho Clinical Diagnostics, *com valor de percentil 99 de 0,034 ng/mL, e ponto de corte para diagnóstico de IAM de 0,12 ng/mL, para sensibilidade de 95% e especificidade de 93% (Figura 1). O CV deste kit no percentil 99 foi <10%, respeitando as recomendações vigentes.^20-22^ As coletas de sangue foram realizadas na admissão no setor de emergência e posteriormente na UCO.

Fonte:TropIES_GEM1309_WW_PT_I_10.pdf. Acessado em http://www.OrthoClinicalDiagnostics.com.

### Delineamento do estudo e análise estatística

Utilizamos os dados de mortalidade e IAM descritos na dissertação de mestrado (https://doi.org/10.11606/D.98.2020.tde-27122019-080250) de estudo conduzido na mesma UCO e estimamos uma diferença relativa de 50% na taxa de eventos entre os grupos com troponina negativa e troponina positiva. Adotando um poder de 90%, alfa de 5%, estimamos 273 casos como tamanho amostral mínimo para os objetivos, com testes de significância bilaterais. O nível de significância adotado foi bilateral < 0,05. As variáveis contínuas foram expressas em média e desvio padrão ou mediana com intervalo interquartil, conforme normalidade da distribuição das variáveis, a qual foi verificada pelo teste de Shapiro-Wilk. As análises de comparações entre os grupos foram efetuadas por o*ne-way* ANOVA ou por método não paramétrico (teste de Kruskal-Wallis). As variáveis categóricas foram expressas como frequências e porcentagens, e comparadas por teste do qui-quadrado ou teste exato de Fisher. A análise dos desfechos foi conduzida conforme o tempo para ocorrência do primeiro evento a partir do início da SCASSST pelo método de Kaplan-Meier com teste de *log-rank* para significância estatística entre as curvas de distribuição de sobrevida para os eventos (óbito, infarto, e desfecho composto). Os programas estatísticos utilizados foram o sistema R e o SPSS Statistics versão 19.0.

## Resultados

### Características dos pacientes e evolução clínica

Avaliamos 494 pacientes com diagnóstico de SCASSST. A tabela 1 apresenta resultados da análise descritiva dos grupos de pacientes. Pacientes com maiores níveis de troponina apresentaram maiores proporções de idosos, maior duração da dor torácica, escores de risco GRACE e CRUSADE mais elevados, menores valores de *clearance* de creatinina e de FEVE, e maior taxa de ocorrência de lesão renal aguda na fase hospitalar (Tabela 1).

**Tabela 1:** – Características clínicas, testes diagnósticos e eventos clínicos dos pacientes divididos em três grupos segundo níveis de troponina

Variável	Troponina				p-valor

	< 0,034 ng/mL		0,034-0,12 ng/mL	> 0,12 ng/mL	
População	122 (24,6%)		63 (12,7%)	309 (62,4%)	-
Sexo masculino	81 (66,4%)		47 (74,6%)	215 (69,6%)	0,47
Idade	63,5 (55-70)		64 (59-71)	66 (59-74)	0,003
Peso	78,5 (69-87,1)		78 (68-87)	75 (66-85)	0,19
Tempo de sintomas	60 (10-292)		80 (15-741)	134 (30-489)	0,019
GRACE na admissão	99 (83-111)		102 (86-122)	120 (103-140)	<0,001
GRACE na alta	84 (70-97)		85 (70-108)	103 (88-120)	<0,001
CRUSADE	26 (19-34)		24 (19-35)	29 (19-40)	0,033
Clear. Creat. (mL/min)	77,5 (69-87)		77 (62-91)	72 (58-87)	0,024
FEVE	59 (50-62)		56 (45-63)	55 (41-60)	0,006
**Antecedentes pessoais**					
AVCi	2 (1,6%)		0	4 (1,3%)	0,42
AVCh	1 (0,8%)		0	0	0,42
CRM	14 (11,4%)		9 (14,2%)	66 (21,3%)	0,03
Dislipidemia	84 (68,8%)		49 (77,7%)	194 (62,7%)	0,05
DAP	3 (2,4%)		4 (6,3%)	20 (6,5%)	0,20
HAS	97 (79,5%)		55 (87,3%)	258 (83,5%)	0,33
IRC	11 (9%)		12 (19%)	69 (22,3%)	<0,01
IAM	60 (49,2%)		28 (44,4%)	172 (55,7%)	0,16
IC	14 (11,4%)		10 (15,8%)	45 (14,6%)	0,61
ICP	43 (35,2%)		20 (31,7%)	103 (33,3%)	0,91
Obesidade	35 (28,6%)		15 (23,8%)	75 (24,3%)	0,65
DM	63 (51,6%)		22 (34,9%)	142 (46%)	0,10
Tabagista	19 (15,5%)		14 (22,2%)	53 (17,2%)	0,71
Ex-tabagista	44 (36%)		24 (38,1%)	119 (38,5%)	0,71
Atividade física	16 (13,1%)		4 (6,3%)	29 (9,4%)	0,34
**Medicações prévias**					
AAS		96 (78,7%)	51 (80,9%)	218 (70,6%)	0,10
Clopidogrel		38 (31,1%)	12 (19%)	89 (28,8%)	0,20
Amiodarona		1 (0,8%)	4 (6,3%)	7 (2,3%)	0,07
BCC		31 (25,4%)	15 (23,8%)	76 (24,6%)	0,98
BRA		50 (40,9%)	28 (44,5%)	109 (35,3%)	0,29
BB oral		80 (65,5%)	40 (63,5%)	208 (67,3%)	0,77
Diurético		39 (31,9%)	26 (41,3%)	112 (36,2%)	0,40
Estatina		92 (75,4%)	48 (76,2%)	218 (70,6%)	0,55
IECA		33 (27%)	18 (28,6%)	111 (35,9%)	0,14
Nitrato		44 (36%)	17 (27%)	101 (32,7%)	0,49
Varfarina		1 (0,8%)	1 (1,6%)	10 (3,2%)	0,36
ADO		53 (43,4%)	16 (25,4%)	120 (38,8%)	0,05
Insulina		22 (18%)	6 (9,5%)	49 (15,9%)	0,33
**Medicações de uso hospitalar**					
AAS		122 (100%)	63 (100%)	309 (100%)	0,37
Clopidogrel		94 (77%)	50 (79,4%)	272 (88%)	<0,01
IECA		61 (50%)	28 (44,4%)	177 (57,3%)	0,09
BRA		49 (40,1%)	24 (38,1%)	89 (28,8%)	0,05
BB oral		112 (91,8%)	55 (87,3%)	290 (93,9%)	0,15
Estatina		122 (100%)	63 (100%)	309 (100%)	0,19
**Classe de Killip-Kimball**					**0,08**
I	117 (95,9%)		60 (95,2%)	265 (85,7%)	
II	5 (4,1%)		2 (3,2%)	32 (10,3%)	
III	0		0	5 (1,6%)	
IV	1 (0,8%)		1 (1,6%)	6 (1,9%)	
**Testes diagnósticos**					
RNM Miocárdica	4 (3,2%)		2 (3,2%)	7 (2,3%)	0,70
Angio TC coronárias	2 (1,6%)		2 (3,2%)	3 (1%)	0,25
Ecocardiograma	102 (83,6%)		51 (81%)	262 (84,8%)	0,61
**Complicações**					
BAV 2° grau	0		1 (1,6%)	2 (0,6%)	0,46
Marcapasso	0		2 (3,2%)	4 (1,3%)	0,14
BIA	2 (1,6%)		1 (1,6%)	4 (1,3%)	1,0
Choque cardiogênico	2 (1,6%)		2 (3,2%)	10 (3,2%)	0,71
LRA	9 (7,4%)		6 (9,5%)	52 (16,8%)	0,01
Hemodiálise	1 (0,8%)		2 (3,2%)	4 (7,5%)	0,10
EAP	1 (0,8%)		0	8 (2,6%)	0,40
FA	6 (4,9%)		3 (4,7%)	20 (6,8%)	0,82
PCR	3 (2,4%)		1 (1,6%)	14 (4,5%)	0,51
Reabordagem cirúrgica	0		0	2 (0,6%)	1,0
Sepse	2 (1,6%)		3 (4,7%)	18 (5,8%)	0,18
TVS	1 (0,8%)		1 (1,6%)	7 (2,3%)	0,78
FV	3 (2,4%)		1 (1,6%)	4 (1,3%)	0,66
Sangramento no sítio de punção	7 (5,7%)		7 (11,2%)	40 (13%)	0,08

*GRACE: Global Registry of Acute Coronary Events; Clear. Creat: clearance de creatinina; FEVE: Fração de ejeção do ventrículo esquerdo; AVCi: acidente vascular cerebral isquêmico; AVCh: acidente vascular cerebral hemorrágico; CRM: cirurgia de revascularização miocárdica; DAP: doença arterial periférica; HAS: hipertensão arterial sistêmica; IRC: insuficiência renal crônica; IAM: infarto agudo do miocárdio; IC: insuficiência cardíaca; ICP: Intervenção Coronária Percutânea; DM: diabetes mellitus; AAS: ácido acetilsalicílico; BCC: bloqueador de canais de cálcio; BRA: bloqueador do receptor de angiotensina II; BB: betabloqueador; IECA: inibidor da enzima conversora de angiotensina; ADO: antidiabéticos orais; RNM: ressonância nuclear magnética; AngioTC: angiotomografia de coronárias; BAV: bloqueio atrioventricular; BIA: balão intra-aórtico; LRA: lesão renal aguda; EAP: edema agudo pulmonar; FA: fibrilação atrial; PCR: parada cardiorrespiratória; TVS: taquicardia ventricular sustentada; FV: fibrilação ventricular.*

### Comparação das estratégias de manejo, desfechos clínicos e revascularização de acordo com os níveis de troponina

Os grupos referentes à troponina elevada tiveram menores proporções de realização de provas funcionais, estratégia invasiva e procedimentos de revascularização miocárdica. Todos os pacientes foram tratados com fármacos de benefício comprovado e recomendações por diretrizes (100% dos pacientes receberam ácido acetilsalicílico e estatinas) (Tabela 1).

**Figura 2:**
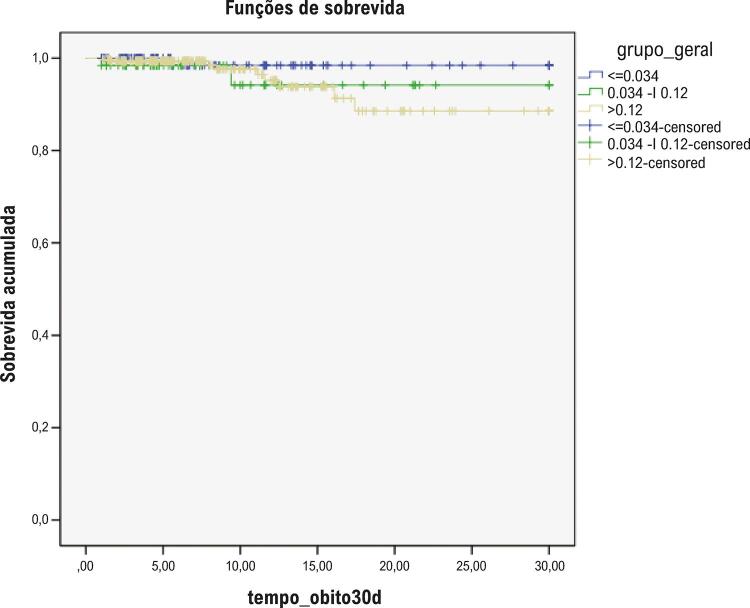
– Curvas de Kaplan-Meier para sobrevida conforme os níveis de troponina (teste de log-rank, p=0,407).

**Figura 3:**
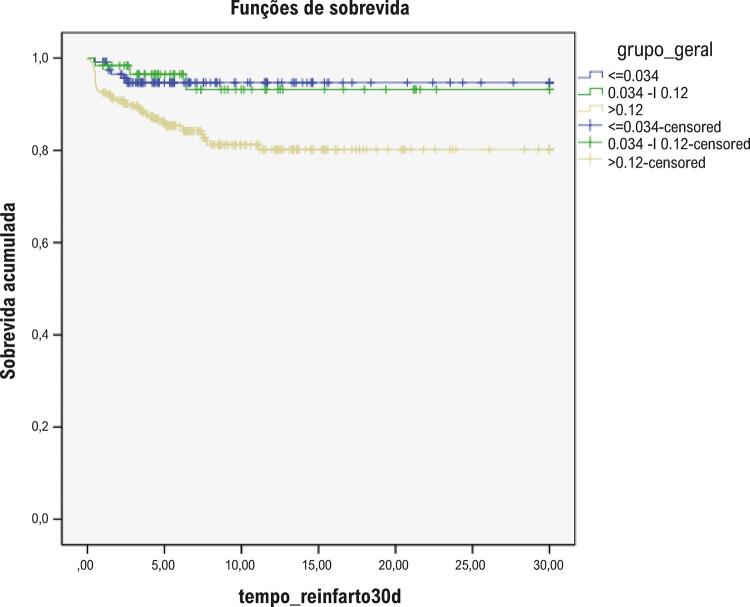
– Curvas de Kaplan-Meier para sobrevida livre de infarto agudo do miocárdio (ou reinfarto) conforme os níveis de troponina (teste de log-rank, p=0,002).

A mortalidade global foi 3,4%, sem diferenças entre os grupos, mas a incidência de IAM (ou reinfarto) foi 2-4 vezes maior no grupo de maior elevação de troponina (Tabela 2). Curvas de Kaplan-Meier para sobrevida global, IAM e desfecho composto estão plotadas, respectivamente, nas Figuras 2, 3 e 4.

**Tabela 2:** – Comparação de estratificação de risco adotada, procedimentos de revascularização e desfechos clínicos entre os grupos de pacientes segundo níveis de troponina

Variáveis	Troponina			p-valor

	< 0,034ng/mL	0,034-0,12ng/mL	> 0,12ng/mL	
População	122 (24,6%)	63 (12,7%)	309 (62,4%)	-
Cintilografia miocárdica	27 (22,1%)	8 (12,7%)	8 (2,6%)	< 0,01
Angiografia coronária	105 (86%)	58 (92,1%)	298 (96,4%)	< 0,01
ICP	35 (28,7%)	29 (46%)	180 (58,3%)	< 0,01
CRM	36 (29,5%)	20 (31,7%)	54 (17,5%)	< 0,01
Nº de enxertos coronários:				< 0,01
1	0	0	3 (1%)	
2	10 (8,2%)	6 (9,5%)	18 (5,8%)	
3	21 (17,2%)	14 (22,3%)	25 (8,1%)	
4	8 (6,5%)	0	8 (2,6%)	
Nº de stents:				
1 stent	25 (20,5%)	25 (39,7%)	118 (38,1%)	0,06
Mais de 1 stent	10 (8,2%)	4 (6,3%)	61 (19,7%)	0,07
Óbito	3 (2,4%)	2 (3,2%)	12 (3,9%)	0,87
IAM (ou Reinfarto)	7 (5,7%)	3 (4,8%)	50 (16,2%)	<0,01

*ICP: intervenção coronária percutânea; CRM: cirurgia de revascularização miocárdica; IAM: infarto agudo do miocárdio.*

**Figura 4:**
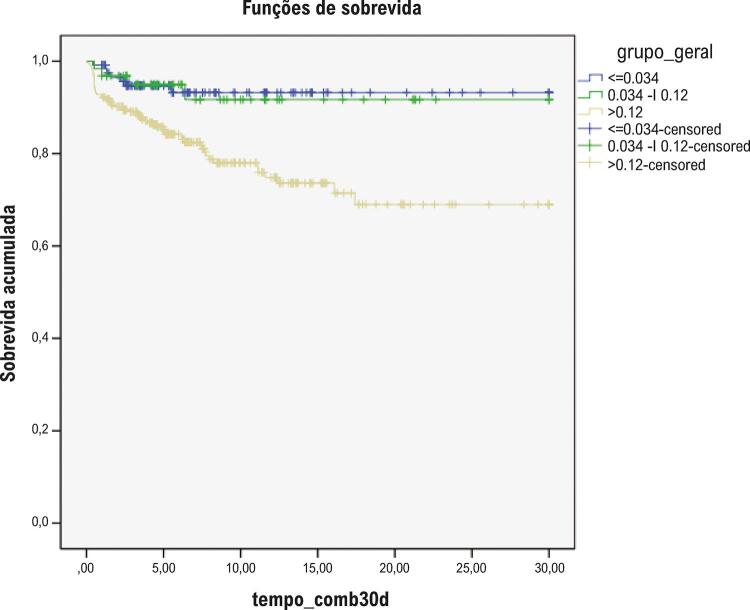
– Curvas de Kaplan-Meier para desfecho composto (óbito ou infarto agudo do miocárdio/reinfarto) conforme os níveis de troponina (teste de log-rank, p<0,001).

## Discussão

A DAC é uma das principais causas de óbito, principalmente quando analisamos o cenário clínico de SCA.^29,30^ Apesar do avanço terapêutico, há elevada morbimortalidade na fase precoce e após alta hospitalar, variando de 5-10% em 30 dias após o evento agudo até 20% em seis meses.^31^ Nesse contexto, diretrizes recomendam a troponina como biomarcador ideal para a estratificação de risco.^32,33^ Pacientes com maiores valores de troponina podem apresentar taxas de 20% de IAM e óbito em 30 dias, e de 25% em seis meses de evolução.^34,35^ Entretanto, diagnóstico diferencial de troponina elevada é essencial e deve ser analisada em conjunto com dados clínicos e exames complementares.^28^

Neste estudo verificamos uma associação entre troponina minimamente elevada e maiores taxas de angiografia coronária e de procedimentos de revascularização. Isto pode ser justificado por esses pacientes apresentarem escores de risco mais elevados, o que aumenta a probabilidade de serem encaminhados para estratégia invasiva durante a hospitalização. Além disso, maiores taxas de IAM foram observadas entre os pacientes com níveis de troponina mais elevados. Este achado apresenta concordância com outros estudos nos quais foi demonstrada a associação do pico da troponina com maiores taxas de eventos adversos.^10-13^ Vale ressaltar a diferença considerável, em número absoluto, de IAM nos pacientes com troponina ≥0,12 ng/mL, mesmo em comparação àqueles com troponina >0,034 ng/mL.

Em termos de mortalidade, apesar de não detectarmos diferença estatisticamente significativa, houve maior número de óbitos nos grupos com troponina elevada, corroborando dados de estudos que evidenciam a relação entre o valor pico da troponina e mortalidade em SCA.^10^ Algumas observações podem explicar este achado. Primeiro, apesar do maior risco de óbito estimado pelo maior escore GRACE, elevação de troponina, gravidade anatômica coronária, e menor FEVE, a maioria dos pacientes dos três grupos foram encaminhados para estratificação invasiva. Assim, não houve associação linear isolada entre maior risco e maior nível de troponina, embora um maior número de pacientes com troponina elevada receberam tratamento de revascularização. Aliada aos fármacos de benefício comprovado na redução de eventos isquêmicos e óbito, essa abordagem mais “invasiva”, baseada não apenas em grandes elevações, mas também em pequenas elevações nos níveis de troponina, pode ter mitigado o desfecho composto que seria esperado pelo maior escore de risco na admissão hospitalar. Portanto, a estratégia invasiva foi relevante para a redução dos eventos cardiovasculares apesar do maior risco inicial. Isso é claramente corroborado pela redução da pontuação obtida no escore de risco GRACE entre a admissão e a alta hospitalar.

Outro dado relevante é a associação entre os dois grupos de troponina elevada, em comparação com pacientes com teste negativo quanto à revascularização miocárdica. De fato, maiores valores de troponina foram associados ao implante de múltiplos *stents* ou maior número de vasos tratados cirurgicamente, o que salienta a plausibilidade biológica entre o pico da troponina e a complexidade anatômica coronária.^8,9^

Por outro lado, embora a Definição Universal de IAM recomende a utilização do percentil 99 para diagnóstico de IAM, ainda há restrições à implementação desse critério pela variabilidade de kits e de valores de referência de troponina I entre os hospitais, podendo interferir na comparabilidade entre estudos clínicos e padronização de protocolos.^25^

Na comparação entre os grupos, embora o número de pacientes submetidos à angiografia coronária não ter sido diferente, a proporção foi significativamente maior conforme o nível de troponina detectado (86% vs 92% vs 96%, respectivamente; p<0,01). Como salientamos acima, a elevada proporção de diagnóstico anatômico realizado por angiografia pode ter influenciado na decisão de proceder à revascularização miocárdica percutânea ou cirúrgica, mesmo no grupo com mínima elevação da troponina, sem significância estatística em relação à mortalidade, apesar da diferença numérica de sobrevida.

Este estudo enfatiza a relevância da utilização do critério diagnóstico de IAM como proposto pela Definição Universal de IAM, especificamente em relação a três aspectos: predição de desfechos cardiovasculares maiores, indicação de estratégia invasiva e realização de procedimentos de revascularização miocárdica, ao compararmos pacientes com diferentes faixas de detecção de troponina I.

O racional da análise de pontos de corte de troponina ocorre devido à grande variabilidade entre hospitais com relação ao nível de troponina considerado para o diagnóstico de IAM, com cerca de 30% de laboratórios hospitalares seguindo as recomendações da Definição Universal de IAM.^14^

Neste estudo, não avaliamos o uso da troponina de alta sensibilidade, pois o objetivo não foi a avaliação da utilidade do biomarcador em protocolos de *rule-in* ou *rule-out* para IAM em sala de emergência, em casos de dor torácica, em pacientes com apresentação muito precoce. De modo oposto, porém complementar à utilidade clínica da troponina, avaliamos o papel prognóstico e a possível influência do ponto de corte na tomada de decisão quanto às estratégias de manejo de SCASSST em unidade de terapia intensiva e o potencial efeito de atenuação do risco precoce estimado por escores, mesmo em casos sem elevação de troponina, em comparação aos pacientes com “mínima” elevação, em decorrência de estratificação invasiva “agressiva” e revascularização apropriada. De fato, nossos dados reforçam a proposição da definição universal de IAM para utilizar o percentil 99 da troponina, minimizando liberações inadvertidas de casos considerados sem elevação da troponina compatível com IAM ao utilizar o ponto de maior acurácia em vez do percentil 99.

### Limitações

O número de eventos observados pode ter reduzido o poder estatístico para detectar diferenças significantes em termos de mortalidade. Entretanto, o elevado índice de angiografia coronária, mesmo nos pacientes sem elevação ou com mínima elevação de troponina, e de subsequente revascularização coronária precoce pode ter reduzido o risco agudo estimado. Por isso, não se pode descartar a hipótese de que não tenha existido diferença na mortalidade pelo fato não ter sido ao acaso. Além disso, os dados foram derivados de único centro, refletindo a prática de uma instituição de ensino e pesquisa, com experiência histórica, alta disponibilidade de exames invasivos e não invasivos, indicadores de desempenho como prescrição de fármacos, *stents* coronários implantados (100% farmacológicos) e uso da artéria torácica interna esquerda, e volume expressivo de procedimentos de intervenção percutânea e revascularização cirúrgica. Tais aspectos poderiam explicar o baixo número de óbitos e IAM mesmo após procedimentos invasivos mais complexos, o que pode não ser aplicável a outros centros com características e infraestruturas diferentes. Ainda, esses fatores podem ter interferido na indicação de angiografia coronária na maioria dos pacientes, sem associação isolada e linear com os níveis de troponina e não necessariamente dependentes de escores de risco mais elevados. Portanto, a associação entre troponina e os desfechos, a estratificação de risco e a indicação de revascularização pode ser diferente em instituições sem disponibilidade de laboratórios de hemodinâmica ou cirurgia cardíaca. Em relação ao escopo do estudo, a grande variedade de kits de troponina I pode influenciar a tomada de decisão local e não ser concordante com nossos achados. Por fim, pela característica exploratória dos estudos observacionais, a variabilidade inerente à seleção de pacientes, e fatores de confusão não mensurados, salientamos que os resultados e conclusões devem ser considerados como indicativos, dando suporte à aplicabilidade clínica em coorte brasileira.

## Conclusões

Valores de troponina I acima do percentil 99 pela Definição Universal de IAM ou acima do ponto de corte de maior acurácia para o diagnóstico de IAM definido para o kit específico apresentam papel prognóstico em termos da ocorrência do desfecho composto de óbito e IAM até 30 dias da SCASSST. De modo mais relevante, níveis de troponina minimamente elevados agregam informação útil ao diagnóstico clínico e escores de risco na tomada de decisão, pela identificação de pacientes com maior probabilidade de benefício com estratificação invasiva e procedimentos de revascularização coronária, o que poderia explicar a atenuação do risco precoce de óbito associado à elevação desse biomarcador.

## References

[1] Schmidt MI, Duncan BB, Silva GA, Menezes AM, Monteiro CA, Barreto SM, et al. Chronic Non-communicable Diseases in Brazil: Burden and Current Challenges. Lancet. 2011;377(9781):1949-61. doi: 10.1016/S0140-6736(11)60135-9. 10.1016/S0140-6736(11)60135-9 21561658

[2] World Health Organization. The World Health Report 2002: Reducing risks, promoting healthy life. Geneva: World Health Organization; 2002.

[3] Dagenais GR, Leong DP, Rangarajan S, Lanas F, Lopez-Jaramillo P, Gupta R, et al. Variations in Common Diseases, Hospital Admissions, and Deaths in Middle-aged Adults in 21 Countries from Five Continents (PURE): A Prospective Cohort Study. Lancet. 2020;395(10226):785-94. doi: 10.1016/S0140-6736(19)32007-0. 10.1016/S0140-6736(19)32007-0 31492501

[4] Badertscher P, Boeddinghaus J, Nestelberger T, Twerenbold R, Wildi K, Sabti Z, et al. Effect of Acute Coronary Syndrome Probability on Diagnostic and Prognostic Performance of High-Sensitivity Cardiac Troponin. Clin Chem. 2018;64(3):515-25. doi: 10.1373/clinchem.2017.279513. 10.1373/clinchem.2017.279513 29343534

[5] Morrow DA, Cannon CP, Rifai N, Frey MJ, Vicari R, Lakkis N, et al. Ability of Minor Elevations of Troponins I and T to Predict Benefit from an Early Invasive Strategy in Patients with Unstable Angina and non-ST Elevation Myocardial Infarction: Results from a Randomized Trial. JAMA. 2001;286(19):2405-12. doi: 10.1001/jama.286.19.2405. 10.1001/jama.286.19.2405 11712935

[6] Rao AC, Collinson PO, Canepa-Anson R, Joseph SP. Troponin T Measurement After Myocardial Infarction can Identify Left Ventricular Ejection of Less Than 40%. Heart. 1998;80(3):223-5. doi: 10.1136/hrt.80.3.223. 10.1136/hrt.80.3.223 PMC1761102 9875077

[7] Sutton MJ, Pfeffer MA, Plappert T, Rouleau JL, Moyé LA, Dagenais GR, et al. Quantitative Two-dimensional Echocardiographic Measurements are Major Predictors of Adverse Cardiovascular Events After Acute Myocardial Infarction. The Protective Effects of Captopril. Circulation. 1994;89(1):68-75. doi: 10.1161/01.cir.89.1.68. 10.1161/01.cir.89.1.68 8281697

[8] Frey N, Dietz A, Kurowski V, Giannitsis E, Tölg R, Wiegand U, et al. Angiographic Correlates of a Positive Troponin T Test in Patients with Unstable Angina. Crit Care Med. 2001;29(6):1130-6. doi: 10.1097/00003246-200106000-00006. 10.1097/00003246-200106000-00006 11395586

[9] Jurlander B, Farhi ER, Banas JJ Jr, Keany CM, Balu D, Grande P, et al. Coronary Angiographic Findings and Troponin T in Patients with Unstable Angina Pectoris. Am J Cardiol. 2000;85(7):810-4. doi: 10.1016/s0002-9149(99)00872-3. 10.1016/s0002-9149(99)00872-3 10758918

[10] Antman EM, Tanasijevic MJ, Thompson B, Schactman M, McCabe CH, Cannon CP, et al. Cardiac-specific Troponin I Levels to Predict the Risk of Mortality in Patients with Acute Coronary Syndromes. N Engl J Med. 1996;335(18):1342-9. doi: 10.1056/NEJM199610313351802. 10.1056/NEJM199610313351802 8857017

[11] Chin CT, Wang TY, Li S, Wiviott SD, deLemos JA, Kontos MC, et al. Comparison of the Prognostic Value of Peak Creatine Kinase-MB and Troponin Levels among Patients with Acute Myocardial Infarction: A Report from the Acute Coronary Treatment and Intervention Outcomes Network Registry-get with the Guidelines. Clin Cardiol. 2012;35(7):424-9. doi: 10.1002/clc.21980. 10.1002/clc.21980 PMC6652484 22434769

[12] Kontos MC, Shah R, Fritz LM, Anderson FP, Tatum JL, Ornato JP, et al. Implication of Different Cardiac Troponin I Levels for Clinical Outcomes and Prognosis of Acute Chest Pain Patients. J Am Coll Cardiol. 2004;43(6):958-65. doi: 10.1016/j.jacc.2003.10.036. 10.1016/j.jacc.2003.10.036 15028350

[13] Diderholm E, Andrén B, Frostfeldt G, Genberg M, Jernberg T, Lagerqvist B, et al. The prognostic and therapeutic implications of increased troponin T levels and ST depression in unstable coronary artery disease: the FRISC II invasive troponin T electrocardiogram substudy. Am Heart J. 2002;143(5):760-7. doi: 10.1067/mhj.2002.121733. 10.1067/mhj.2002.121733 12040335

[14] Bagai A, Huang Z, Lokhnygina Y, Harrington RA, Armstrong PW, Strony J, et al. Magnitude of Troponin Elevation and Long-term Clinical Outcomes in Acute Coronary Syndrome Patients Treated with and without Revascularization. Circ Cardiovasc Interv. 2015;8(6):e002314. doi: 10.1161/CIRCINTERVENTIONS.115.002314. 10.1161/CIRCINTERVENTIONS.115.002314 26025218

[15] Bhatt HA, Sanghani DR, Lee D, Julliard KN, Fernaine GA. Predictors of Peak Troponin Level in Acute Coronary Syndromes: Prior Aspirin Use and SYNTAX Score. Int J Angiol. 2016;25(1):54-63. doi: 10.1055/s-0035-1547396. 10.1055/s-0035-1547396 PMC4758844 26900312

[16] Castro LT, Santos IS, Goulart AC, Pereira ADC, Staniak HL, Bittencourt MS, et al. Elevated High-Sensitivity Troponin I in the Stabilized Phase after an Acute Coronary Syndrome Predicts All-Cause and Cardiovascular Mortality in a Highly Admixed Population: A 7-Year Cohort. Arq Bras Cardiol. 2019;112(3):230-7. doi: 10.5935/abc.20180268. 10.5935/abc.20180268 PMC6424029 30916200

[17] Cannon CP, Weintraub WS, Demopoulos LA, Vicari R, Frey MJ, Lakkis N, et al. Comparison of Early Invasive and Conservative Strategies in Patients with Unstable Coronary Syndromes Treated with the glycoprotein IIb/IIIa Inhibitor Tirofiban. N Engl J Med. 2001;344(25):1879-87. doi: 10.1056/NEJM200106213442501. 10.1056/NEJM200106213442501 11419424

[18] Fuchs S, Kornowski R, Mehran R, Satler LF, Pichard AD, Kent KM, et al. Cardiac Troponin I Levels and Clinical Outcomes in Patients with Acute Coronary Syndromes: The Potential Role of Early Percutaneous Revascularization. J Am Coll Cardiol. 1999;34(6):1704-10. doi: 10.1016/s0735-1097(99)00434-9. 10.1016/s0735-1097(99)00434-9 10577560

[19] Romano ER. Elaboração de um Escore de Risco para Síndrome Coronária Aguda em Hospital Terciário Privado [dissertation]. São Paulo: Instituto Dante Pazzanese de Cardiologia; 2013.

[20] Apple FS, Jesse RL, Newby LK, Wu AH, Christenson RH. National Academy of Clinical Biochemistry and IFCC Committee for Standardization of Markers of Cardiac Damage Laboratory Medicine Practice Guidelines: Analytical issues for biochemical markers of acute coronary syndromes. Circulation. 2007;115(13):352-5. doi: 10.1161/CIRCULATIONAHA.107.182881. 10.1161/CIRCULATIONAHA.107.182881 17384332

[21] Apple FS. A New Season for Cardiac Troponin Assays: It’s Time to Keep a Scorecard. Clin Chem. 2009;55(7):1303-6. doi: 10.1373/clinchem.2009.128363. 10.1373/clinchem.2009.128363 19478023

[22] Apple FS, Parvin CA, Buechler KF, Christenson RH, Wu AH, Jaffe AS. Validation of the 99th Percentile Cutoff Independent of Assay Imprecision (CV) for Cardiac Troponin Monitoring for Ruling out Myocardial Infarction. Clin Chem. 2005;51(11):2198-200. doi: 10.1373/clinchem.2005.052886. 10.1373/clinchem.2005.052886 16244304

[23] Agirbasli M. Universal Definition of MI: Above 99 Percentile of Upper Reference Limit (URL) for hs-cTn: Yes, but Which URL? Am J Emerg Med. 2019;37(3):510. doi: 10.1016/j.ajem.2018.12.052. 10.1016/j.ajem.2018.12.052 30600186

[24] Sandoval Y, Apple FS. The Global Need to Define Normality: The 99th Percentile Value of Cardiac Troponin. Clin Chem. 2014;60(3):455-62. doi: 10.1373/clinchem.2013.211706. 10.1373/clinchem.2013.211706 24115136

[25] Bagai A, Alexander KP, Berger JS, Senior R, Sajeev C, Pracon R, et al. Use of Troponin Assay 99th Percentile as the Decision Level for Myocardial Infarction Diagnosis. Am Heart J. 2017;190:135-9. doi: 10.1016/j.ahj.2017.04.016. 10.1016/j.ahj.2017.04.016 PMC5543710 28760208

[26] Apple FS, Sandoval Y, Jaffe AS, Ordonez-Llanos J. Cardiac Troponin Assays: Guide to Understanding Analytical Characteristics and Their Impact on Clinical Care. Clin Chem. 2017;63(1):73-81. doi: 10.1373/clinchem.2016.255109. 10.1373/clinchem.2016.255109 28062612

[27] Thygesen K, Alpert JS, White HD, Jaffe AS, Apple FS, Galvani M, et al. Universal Definition of Myocardial Infarction. Circulation. 2007;116(22):2634-53. doi: 10.1161/CIRCULATIONAHA.107.187397. 10.1161/CIRCULATIONAHA.107.187397 17951284

[28] Roffi M, Patrono C, Collet JP, Mueller C, Valgimigli M, Andreotti F, et al. 2015 ESC Guidelines for the Management of Acute Coronary Syndromes in Patients Presenting without Persistent ST-Segment Elevation: Task Force for the Management of Acute Coronary Syndromes in Patients Presenting without Persistent ST-Segment Elevation of the European Society of Cardiology (ESC). Eur Heart J. 2016;37(3):267-315. doi: 10.1093/eurheartj/ehv320. 10.1093/eurheartj/ehv320 26320110

[29] Bassan R, Pimenta L, Leães PE, Timerman A, Volschan A, Polanczyk C, et al. I Diretriz de Dor Torácica na Sala de Emergência. Arq. Bras. Cardiol. 2002;79(Suppl 2):1-22. doi: 10.1590/S0066-782X2002001700001. 12386722

[30] Tabnet. DataSUS. Sistema de Informações Hospitalares do SUS (SIH/SUS). Brasília: Ministério da Saúde; c2022 [cited 2022 Feb 02]. Available from: http://tabnet.datasus.gov.br.2004.

[31] Grech ED, Ramsdale DR. Acute Coronary Syndrome: Unstable Angina and non-ST Segment Elevation Myocardial Infarction. BMJ. 2003;326(7401):1259-61. doi: 10.1136/bmj.326.7401.1259. 10.1136/bmj.326.7401.1259 PMC1126130 12791748

[32] Jaffe AS, Ravkilde J, Roberts R, Naslund U, Apple FS, Galvani M, et al. It’s Time for a Change to a Troponin Standard. Circulation. 2000;102(11):1216-20. doi: 10.1161/01.cir.102.11.1216. 10.1161/01.cir.102.11.1216 10982533

[33] Ohman EM, Armstrong PW, Christenson RH, Granger CB, Katus HA, Hamm CW, et al. Cardiac Troponin T Levels for Risk Stratification in Acute Myocardial Ischemia. GUSTO IIA Investigators. N Engl J Med. 1996;335(18):1333-41. doi: 10.1056/NEJM199610313351801. 10.1056/NEJM199610313351801 8857016

[34] Zimmerman J, Fromm R, Meyer D, Boudreaux A, Wun CC, Smalling R, et al. Diagnostic Marker Cooperative Study for the Diagnosis of Myocardial Infarction. Circulation. 1999;99(13):1671-7. doi: 10.1161/01.cir.99.13.1671. 10.1161/01.cir.99.13.1671 10190875

[35] Polanczyk CA, Lee TH, Cook EF, Walls R, Wybenga D, Printy-Klein G, et al. Cardiac Troponin I as a Predictor of Major Cardiac Events in Emergency Department Patients with Acute Chest Pain. J Am Coll Cardiol. 1998;32(1):8-14. doi: 10.1016/s0735-1097(98)00176-4. 10.1016/s0735-1097(98)00176-4 9669242

