# Supplementary material for: Troponina I por Percentil 99 da Definição Universal de Infarto do Miocárdio versus Ponto de Corte de Melhor Acurácia em Síndromes Coronárias Agudas
Source: Arq Bras Cardiol. 2022 Apr 26;118(6):1006–15. [Article in Portuguese] doi: 10.36660/abc.20210191 (PMC9345147; doi:10.36660/abc.20210191)
Supplement: Translation

# Translation: Universal Definition of Myocardial Infarction 99^th^ Percentile versus Diagnostic Cut-off Value of Troponin I for Acute Coronary Syndromes

**Authors:** Antonio Haddad Tapias, Gustavo Bernardes de Figueiredo Oliveira, João Italo Dias França, Rui Fernando Ramos

**Affiliations:** 1 Brazil Instituto Dante Pazzanese de Cardiologia, São Paulo, SP – Brazil

**Keywords:** Troponin I, Acute Coronary Syndrome, Myocardial Revascularization

## Abstract

**Background:**

Contemporary diagnosis of ACS and risk stratification are essential for appropriate management and reduction of mortality and recurrent ischemic events, in the acute phase of disease and after hospitalization. The Universal Definition of Myocardial Infarction recommends the detection of troponin levels above the 99^th^ percentile.

**Objectives:**

To evaluate the occurrence of early death and acute myocardial infarction (AMI) in patients without elevation of troponin (<0.034 ng/mL), patients with mild elevation (above the 99^th^ percentile [>0.034 ng/mL and <0.12 ng/mL)], and patients with significant elevation of troponin (above the diagnostic cutoff for AMI defined by the troponin kit (≥0.12 ng/mL)]; and to analyze the impact of troponin on the indication for invasive strategy and myocardial revascularization.

**Methods:**

Cross-sectional cohort study of patients with ACS with assessment of peak troponin I, risk score, prospective analysis of 30-day clinical outcomes and two-sided statistical tests, with statistical significance set at p<0.05.

**Results:**

A total of 494 patients with ACS were evaluated. Troponin > 99^th^ percentile and below the cutoff point, as well as values above the cutoff, were associated with higher incidence of composite endpoint (p<0.01) and higher rates of percutaneous or surgical revascularization procedures (p<0.01), without significative difference in 30-day mortality.

**Conclusions:**

Troponin levels above the 99^th^ percentile defined by the universal definition of AMI play a prognostic role and add useful information to the clinical diagnosis and risk scores by identifying those patients who would most benefit from invasive risk stratification and coronary revascularization procedures.

## Introduction

Cardiovascular diseases are the main cause of death among people older than 60 years in Brazil,^1^ and important causes of disability, hospitalizations and death, mainly in low per capita income countries.^2,3^

In acute coronary syndromes (ACS), serial electrocardiography (ECG) and troponin measurements, associated with clinical examination, are essential for the correct diagnosis and appropriate management of disease.^4^ In the context of acute myocardial ischemia, in addition to establishing the diagnosis of acute myocardial infarction (AMI), the measurement of troponin is useful for risk stratification in invasive strategies.^5^ Besides, peak troponin levels are correlated with the extension of necrosis and left ventricular ejection fraction (LVEF), which are important determinants of post-AMI mortality.^6,7^ In addition, elevated troponin levels have been correlated with multivessel coronary artery disease (CAD) and greater severity of stenosis in patients with non-ST-elevation acute coronary syndrome (NSTE-ACS),^8,9^ in addition to a directly proportional relationship with rates of clinical outcomes^10-13^ in patients undergoing early revascularization procedures.^14^

Factors including severity and complexity of CAD, previous use of acetylsalicylic acid and early coronary angiography are associated with peak troponin in NSTE-ACS.^15^ Among patients stabilized after an ACS, increased troponin is associated with higher all-cause cardiovascular mortality, regardless of covariables.^16^ Individuals with very high levels of troponin have more complex CAD and, based on pathophysiological plausibility, revascularization may be more often indicated in this group as compared with patients without troponin elevation. On the other hand, some authors have not found an association between high troponin levels and worse clinical outcomes.^17,18^

### Troponin levels proposed for the diagnosis of ACS

According to the International Federation of Clinical Chemistry and the National Academy of Clinical Biochemistry, increased troponin levels are defined as those above the 99^th^ percentile of a healthy population, and an intra-assay coefficient of variation (CV) <10%,^19^ although many troponin test kits have poor accuracy based on this percentile.^20-22^ Therefore, for the use of troponin in the diagnosis of AMI, it is necessary an ascending or descending curve of the biomarker, including at least one value above the 99^th^ percentile of the reference population, according to sex, ethnics and other factors.^23,24^ Some studies have pointed out the importance of using standard levels for the diagnosis of AMI in hospital laboratories to improve clinical decisions, to tailor diagnostic thresholds to the population seen in each institution, and facilitate reporting in clinical trials.^25,26^

Therefore, the present study aimed to evaluate the occurrence of clinically relevant outcomes (death, AMI, and composite endpoint) in patients at the early stage of NSTE-ACS; to compare three groups formed according to the ranges of troponin I values – without elevation (<0.034 ng/mL, i.e., below the 99^th^ percentile), mild increase (Universal Definition of AMI, above the 99^th^ percentile [>0.034 ng/mL and <0.12 ng/mL]), and significant increase (the most accurate diagnostic cut-off defined by local troponin kit [≥0.12 ng/mL]; and to assess the association between these groups and requirement of an invasive strategy or myocardial revascularization procedures during hospital stay. The hypothesis is that the 99^th^ percentile value of troponin, even if lower than the cut-off point for troponin I defined by the commercial kit, is associated with clinical impact and indication for invasive stratification and myocardial revascularization, in comparison with negative levels, corroborating the values proposed by the Universal Definition of AMI.

## Methods

### Characteristics of the study and ethical aspects

Observational, cross-sectional study, with follow-up of up to 30 days for evaluation of death and infarction rates, and composite endpoint in patients with NSTE-ACS admitted to a coronary care unit (CCU), divided into groups according to troponin levels. All clinical events were pre-defined and assessed following a systematic collection of data from databases. Indication for invasive or non-invasive risk stratification, in-hospital treatments, and routine laboratory tests were also evaluated. All participants signed an informed consent form. The study was approved by local ethics committee in April 2019. Recruitment of patients was carried out from May 2019 to January 2020.

### Inclusion criteria

•  Age ≥ 18 years
•  Admission to a CCU
•  Diagnosis of NSTE-ACS
•  The diagnosis of NSTE-ACS was made based on two of the following criteria:
- Clinical presentation suggestive of ACS;
- ECG showing depression of the ST segment, T-wave inversion or non-specific findings;
- Ascending or descending troponin curves, including at least one value above 0.12 ng/mL (diagnostic value for AMI in the troponin I kit used at Dante Pazzanese Institute of Cardiology).

Reinfarction was defined following the recommendations of the fourth universal definition of AMI, and considered suspected in the presence of signs or symptoms of infarction, requiring another troponin measurement in this case. Diagnosis is confirmed by a 20% increase in troponin levels in patients with already elevated values, or a new increase in those with previously normal levels.

•  The diagnosis of unstable angina was made based on two of the following criteria:
- Clinical presentation suggestive of ACS;
- ECG showing depression of the ST segment, T-wave inversion or non-specific findings;
- Absence of troponin levels above 0.034 ng/mL (according to troponin levels for the diagnosis of AMI proposed by the universal definition of AMI^27^ and the European Society of Cardiology guidelines^28^).

### Exclusion criteria

- Absence of consent to participate in the study
- Patients referred for invasive management 48 hours after the first episode of ACS.

### Variables analyzed

Demographic data, cardiovascular risk factors, comorbidities, previous use of medications, non-invasive hemodynamic parameters, coronary angiographic findings (of patients referred for invasive strategy), GRACE and CRUSADE scores were analyzed. Laboratory tests, therapeutic procedures and approaches during hospital stay were performed according to institutional protocols.

### Cardiac troponin test

The VITROS® high-sensitivity troponin I assay (Ortho Clinical Diagnostics) was used for measurements of cardiac troponin I, with a 99^th^ percentile value of 0.034 ng/mL, diagnostic cut off point of 0.12 ng/mL for AMI, sensitivity of 95% and specificity of 93% (Figure 1). The CV of the kit at the 99^th^ percentile was <10%, according to current recommendations.^20-22^ Blood collection was performed at admission to the emergency department and at the CCU.

**Figure 1:** – Specifications of the VITROS® high-sensitivity troponin I assay.

Fonte:TropIES_GEM1309_WW_PT_I_10.pdf. Acessado em http://www.OrthoClinicalDiagnostics.com.

### Study design and statistical analysis

We used the mortality and AMI data described on a Masters thesis of a study conducted at the same CCU (available at: https://doi.org/10.11606/D.98.2020.tde-27122019-080250), and estimated a relative difference of 50% in the rate of events between the groups with negative and positive troponin. Using a power of 90% and alpha of 5%, we estimated a minimum sample size of 273 patients for the objectives of the study. Two-sided significance tests were used, with significance level at 0.05. Continuous variables were expressed as mean and standard deviation or median and interquartile range, according to normality of distributions, which was tested using the Shapiro-Wilk test. Between-group comparisons were assessed by the one-way ANOVA or by the non-parametric Kruskal-Wallis test. Categorical variables were expressed as frequency and percentages and compared by the chi-square test or Fisher exact test. Analysis of outcomes was conducted according to the time to the first event since the onset of NSTE-ACS by the Kaplan-Meier method and the log-rank test for statistical significance between survival curves for the events (death, infarction, and composite endpoint). Statistical analysis was performed using the R system and the SPSS statistics, version 19.0.

## Results

### Patients’ characteristics and clinical course

A total of 494 patients with diagnosis of NSTE-ACS were evaluated. Table 1 presents the results of the descriptive analysis of the groups. The group of patients with significant increase in troponin levels had a higher proportion of older people, longer duration of chest pain, higher GRACE and CRUSADE scores, lower creatinine clearance levels, lower LVEF, and higher rates of acute kidney injury during hospitalization (Table 1).

**Table 1:** – Clinical characteristics, diagnostic tests and clinical events of the study population divided into three groups by troponin levels

Variable	Troponin				p-value

	< 0.034 ng/mL		0.034-0.12 ng/mL	> 0.12 ng/mL	
Population	122 (24.6%)		63 (12.7%)	309 (62.4%)	-
Male sex	81 (66.4%)		47 (74.6%)	215 (69.6%)	0.47
Age	63.5 (55-70)		64 (59-71)	66 (59-74)	0.003
Weight	78.5 (69-87.1)		78 (68-87)	75 (66-85)	0.19
Duration of symptoms	60 (10-292)		80 (15-741)	134 (30-489)	0.019
GRACE at admission	99 (83-111)		102 (86-122)	120 (103-140)	<0.001
GRACE at discharge	84 (70-97)		85 (70-108)	103 (88-120)	<0.001
CRUSADE	26 (19-34)		24 (19-35)	29 (19-40)	0.033
Creat. Clear. (mL/min)	77.5 (69-87)		77 (62-91)	72 (58-87)	0.024
LVEF	59 (50-62)		56 (45-63)	55 (41-60)	0.006
**Clinical history**					
Ischemic stroke	2 (1.6%)		0	4 (1.3%)	0.42
Hemorrhagic stroke	1 (0.8%)		0	0	0.42
CABG	14 (11.4%)		9 (14.2%)	66 (21.3%)	0.03
Dyslipidemia	84 (68.8%)		49 (77.7%)	194 (62.7%)	0.05
PAD	3 (2.4%)		4 (6.3%)	20 (6.5%)	0.20
Hypertension	97 (79.5%)		55 (87.3%)	258 (83.5%)	0.33
CRF	11 (9%)		12 (19%)	69 (22.3%)	<0.01
AMI	60 (49.2%)		28 (44.4%)	172 (55.7%)	0.16
HF	14 (11.4%)		10 (15.8%)	45 (14.6%)	0.61
PCI	43 (35.2%)		20 (31.7%)	103 (33.3%)	0.91
Obesity	35 (28.6%)		15 (23.8%)	75 (24.3%)	0.65
DM	63 (51.6%)		22 (34.9%)	142 (46%)	0.10
Smoker	19 (15.5%)		14 (22.2%)	53 (17.2%)	0.71
Former smoker	44 (36%)		24 (38.1%)	119 (38.5%)	0.71
Physical activity	16 (13.1%)		4 (6.3%)	29 (9.4%)	0.34
**Previous medications **					
ASA		96 (78.7%)	51 (80.9%)	218 (70.6%)	0.10
Clopidogrel		38 (31.1%)	12 (19%)	89 (28.8%)	0.20
Amiodarone		1 (0.8%)	4 (6.3%)	7 (2.3%)	0.07
CCB		31 (25.4%)	15 (23.8%)	76 (24.6%)	0.98
ARB		50 (40.9%)	28 (44.5%)	109 (35.3%)	0.29
Oral BB		80 (65.5%)	40 (63.5%)	208 (67.3%)	0.77
Diuretics		39 (31.9%)	26 (41.3%)	112 (36.2%)	0.40
Statin		92 (75.4%)	48 (76.2%)	218 (70.6%)	0.55
ACEi		33 (27%)	18 (28.6%)	111 (35.9%)	0.14
Nitrates		44 (36%)	17 (27%)	101 (32.7%)	0.49
Warfarin		1 (0.8%)	1 (1.6%)	10 (3.2%)	0.36
OADs		53 (43.4%)	16 (25.4%)	120 (38.8%)	0.05
Insulin		22 (18%)	6 (9.5%)	49 (15.9%)	0.33
**Medications during hospitalization **					
ASA		122 (100%)	63 (100%)	309 (100%)	0.37
Clopidogrel		94 (77%)	50 (79.4%)	272 (88%)	<0.01
ACEi		61 (50%)	28 (44.4%)	177 (57.3%)	0.09
ARB		49 (40.1%)	24 (38.1%)	89 (28.8%)	0.05
Oral BB		112 (91.8%)	55 (87.3%)	290 (93.9%)	0.15
Statin		122 (100%)	63 (100%)	309 (100%)	0.19
**Killip class**					**0.08**
I	117 (95.9%)		60 (95.2%)	265 (85.7%)	
II	5 (4.1%)		2 (3.2%)	32 (10.3%)	
III	0		0	5 (1.6%)	
IV	1 (0.8%)		1 (1.6%)	6 (1.9%)	
**Diagnostic tests**					
Cardiac MRI	4 (3.2%)		2 (3.2%)	7 (2.3%)	0.70
CCTA	2 (1.6%)		2 (3.2%)	3 (1%)	0.25
Echocardiography	102 (83.6%)		51 (81%)	262 (84.8%)	0.61
**Complications**					
Second degree-AVB	0		1 (1.6%)	2 (0.6%)	0.46
Pacemaker	0		2 (3.2%)	4 (1.3%)	0.14
IAB	2 (1.6%)		1 (1.6%)	4 (1.3%)	1.0
Cardiogenic shock	2 (1.6%)		2 (3.2%)	10 (3.2%)	0.71
AKI	9 (7.4%)		6 (9.5%)	52 (16.8%)	0.01
Hemodialysis	1 (0.8%)		2 (3.2%)	4 (7.5%)	0.10
APE	1 (0.8%)		0	8 (2.6%)	0.40
AF	6 (4.9%)		3 (4.7%)	20 (6.8%)	0.82
CRA	3 (2.4%)		1 (1.6%)	14 (4.5%)	0.51
Second surgical approach	0		0	2 (0.6%)	1.0
Sepsis	2 (1.6%)		3 (4.7%)	18 (5.8%)	0.18
SVT	1 (0.8%)		1 (1.6%)	7 (2.3%)	0.78
VF	3 (2.4%)		1 (1.6%)	4 (1.3%)	0.66
Punction-site bleeding	7 (5.7%)		7 (11.2%)	40 (13%)	0.08

*GRACE: Global Registry of Acute Coronary Events; CC: creatinine clearance; LVEF: left ventricular ejection fraction; CABG: coronary artery bypass grafting; PAD: peripheral arterial disease; CRF: chronic renal failure; AMI: acute myocardial infarction; HF: heart failure; PCI: percutaneous coronary intervention; DM: diabetes mellitus; ASA: acetylsalicylic acid; CCB: calcium channel blockers; ARB: angiotensin II receptor blocker; BB: beta-blocker; ACEI: angiotensin converting enzyme inhibitors; OAD: oral antidiabetics; MRI: magnetic resonance imaging; CCTA: coronary computed tomography angiography; AVB: atrioventricular block; IAB: intra-aortic balloon; AKI: acute kidney injury; APE: acute pulmonary edema; AF: atrial fibrillation; CRA: cardiorespiratory arrest; SVT: sustained ventricular tachycardia; VF: ventricular fibrillation.*

### Comparison of management strategies, clinical outcomes, and revascularization according to troponin levels

A greater number of patients with significant increase in troponin levels underwent functional studies, invasive approach, and myocardial revascularization procedures. All patients were treated with benefit-proven medications recommended by guidelines (100% of patients received acetylsalicylic acid and statins) (Table 1).

Overall mortality was 3.4%, with no statistically significant difference between the groups, although the incidence of AMI (or reinfarction) was 2-4 times higher in the group with troponin elevation (Table 2). Kaplan–Meier plots depicting overall survival, AMI and composite endpoint are illustrated in Figures 2, 3 and 4, respectively.

**Table 2:** – Comparison of risk stratification, revascularization procedures and clinical outcomes between the groups of patients by troponin levels

Variables	Troponin			p-value

	< 0.034	0.034-0.12	> 0.12	
Population	122 (24.6%)	63 (12.7%)	309 (62.4%)	-
Myocardial scintigraphy	27 (22.1%)	8 (12.7%)	8 (2.6%)	< 0.01
Coronary angiography	105 (86%)	58 (92.1%)	298 (96.4%)	< 0.01
PCI	35 (28.7%)	29 (46%)	180 (58.3%)	< 0.01
CABG	36 (29.5%)	20 (31.7%)	54 (17.5%)	< 0.01
Number of coronary grafts:				< 0.01
1	0	0	3 (1%)	
2	10 (8.2%)	6 (9.5%)	18 (5.8%)	
3	21 (17.2%)	14 (22.3%)	25 (8.1%)	
4	8 (6.5%)	0	8 (2.6%)	
**Number of stents:**				
1 stent	25 (20.5%)	25 (39.7%)	118 (38.1%)	0.06
> 1 stent	10 (8.2%)	4 (6.3%)	61 (19.7%)	0.07
Death	3 (2.4%)	2 (3.2%)	12 (3.9%)	0.87
AMI (or reinfarction)	7 (5.7%)	3 (4.8%)	50 (16.2%)	<0.01

*PCI: percutaneous coronary intervention; CABG: coronary artery bypass grafting; AMI: acute myocardial infarction*

**Figure 2:** – Kaplan Meier curves illustrating event-free survival after acute myocardial infarction (or reinfarction) by troponin range groups (log-rank test, p=0.002).

**Figure 3:** – Kaplan Meier curves illustrating event-free survival after acute myocardial infarction (or reinfarction) by troponin range groups (log-rank test, p=0.002).

**Figure 4:** – Kaplan-Meier curves of composite endpoint (death or acute myocardial infarction/reinfarction) by troponin range groups (log-rank test p<0.001).

## Discussion

CAD is one of the main causes of death, especially in the context of ACS.^29,30^ Despite therapeutic advances, morbidity and mortality of CAD in the early stages of disease and after hospital discharge are high, varying from 5-10% within 30 days to 20% in six months after the acute event.^31^ In this context, guidelines have recommended the use of troponin as a biomarker for risk stratification.^32,33^ Patients with increased troponin may have a 20% rate of AMI and death in 30 days, and a 25% rate in six months of follow-up.^34,35^ However, differential diagnosis by increased troponin is crucial and should be analyzed together with clinical data and complementary tests.^28^

In the present study, we observed an association between mild elevations of troponin and higher rates of coronary angiography and revascularization procedures. This may be explained by the fact that these patients had higher risk scores, which increases the likelihood of referral for invasive procedures during hospitalization. Also, higher rates of AMI were found in patients with increased troponin. This is in agreement with previous studies showing that peak troponin values are associated with higher rates of adverse events.^10-13^ It is worth mentioning the considerably higher number of cases of AMI among patients with troponin levels ≥0.12 ng/mL, even as compared with those with troponin > 0.034 ng/mL.

Regarding mortality rates, although we have not detected statistically significant difference, a higher number of deaths was found among patients with elevated troponin, corroborating other studies that showed a relationship between peak troponin values and mortality in ACS.^10^ These data may be explained by some factors. First, despite the higher risk of death estimated by higher GRACE score, troponin elevation, severity of CAD, and lower LVEF, most patients of the three groups were referred for invasive risk stratification. Therefore, there was not a linear association between increased risk and elevated troponin, although a larger number of patients with a marked increase in troponin levels underwent revascularization. Combined with the use of medications with proven benefits in reducing ischemic events and death, this “more invasive” approach, based not only on significant elevations but also on mild elevations in troponin levels, may have mitigated the occurrence of composite events that would be expected due to high risk score at admission. Therefore, the invasive strategy was important to reduce cardiovascular events despite increased initial risk. This is clearly corroborated by the reduction in GRACE score from hospital admission to discharge.

Another important finding is the association between the two groups of patients with elevated troponin in comparison with patients with normal levels regarding myocardial revascularization. Higher troponin levels were associated with multiple stent implantation and higher number of surgically treated vessels, which reinforces the biological plausibility linking increased troponin levels with complexity of coronary anatomy.^8,9^

On the other hand, although the universal definition of AMI recommends the use of the 99^th^ percentile for the diagnosis of AMI, its widespread implementation is still hampered by between-kit variability and variation of troponin I reference values between hospitals, which may influence the comparability between clinical trials and standardization of protocols.^25^

In the comparison between the groups, although the number of patients who underwent coronary angiography was not different, the proportion of patients was significantly higher in those with greater elevation of troponin (86% vs 92% vs 96%, respectively; p<0.01). As we pointed out, the high proportion of anatomic diagnosis made by angiography may have influenced the decision to perform percutaneous or surgical myocardial revascularization, even in patients with mild increase of troponin, with no statistical difference regarding mortality, despite numerical difference in survival.

This study emphasizes the importance of using the diagnostic criteria of AMI proposed by the universal definition of AMI, particularly with respect to three aspects – prediction of major cardiovascular outcomes, indication of invasive strategy, and performance of myocardial revascularization procedures in the comparison of groups of patients with different troponin levels.

The rationale of the analysis of troponin cutoff points is the high variability of the diagnostic value between the hospitals, with nearly 30% of hospital laboratories following the recommendations by the universal definition of AMI.^14^

In the present study, we did not evaluate the use of high-sensitivity troponin since our objective was not to analyze the usefulness of this biomarker in rule-in or rule-out protocols for AMI in the emergency room in cases of chest pain, in patients with very early presentation. However, we did analyze the prognostic role and the possible influence of troponin cutoff values on the decision making in the management of NSTE-ACS in intensive care unit as well as the potential effect of attenuating the early risk (estimated by scores), even in patients without troponin elevation, as compared with patients with slight elevation, by an “aggressive” invasive risk stratification and appropriate revascularization. Indeed, our data reinforce the use of the 99^th^ percentile concentration of cardiac troponin proposed by the universal definition of AMI, avoiding unnecessary dismissal of patients without troponin elevations compatible with AMI when adopting a more accurate cutoff instead of the 99^th^ percentile.

### Limitations

The number of events observed may have reduced the statistical power to detect significant differences in terms of mortality. However, the high rate of coronary angiography, even among patients with no or minimal elevation of troponin, and subsequent early coronary revascularization may have reduced the estimated acute risk. Therefore, the hypothesis of a difference in mortality cannot be excluded, as this was not a random situation. Second, our data derived from a single center and reflected the reality of a research and education institution, with historical experience, and where invasive and non-invasive tests, indicators of performance such as drug prescription, coronary stent implantation (100% drug-eluting stents) with use of the left internal thoracic artery are highly available, and a large volume of percutaneous intervention and surgical revascularization has been performed. These factors could probably explain the low number of deaths and AMI even after highly complex invasive procedures, which may not be applied to other centers with different characteristics and infrastructures. Also, these aspects may have influenced the indication for coronary angiography in the majority of patients, without an isolated and linear association with troponin levels, and not necessarily dependent on more elevated risk scores. Thus, the association of troponin with outcomes, risk stratification and indication of revascularization may be different in institutions where hemodynamic laboratories and cardiac surgery are not available. Considering the scope of the study, the high variability of troponin I kits may influence local decisions and produce divergent results. Finally, due to the exploratory nature of observational studies, variability inherent to the selection of patients, and unmeasured confounding factors, we emphasize that the results and conclusions obtained in this study should be considered just an indication and be used as a support to their applicability in Brazilian populations.

## Conclusions

Cardiac troponin values above the 99^th^ percentile, proposed by the universal definition of AMI, or above the most accurate diagnostic cut-off point for AMI, defined by specific kit, have prognostic value in terms of the occurrence of composite endpoint of death and AMI within 30 days after NSTE-ACS. More importantly, mild elevations of troponin add useful information to the clinical diagnosis and risk scores in the decision-making process, by identifying those patients who would most benefit from invasive risk stratification and coronary revascularization procedures, which could explain the attenuation of early mortality risk associated with the increase in this biomarker.
